# Oral Vaccination with Engineered Probiotic Limosilactobacillus reuteri Has Protective Effects against Localized and Systemic Staphylococcus aureus Infection

**DOI:** 10.1128/spectrum.03673-22

**Published:** 2023-02-01

**Authors:** Na Pan, Yang Liu, Haochi Zhang, Ying Xu, Xuemei Bao, Shouxin Sheng, Yanchen Liang, Bohui Liu, Yueqing Lyu, Haotian Li, Fangfei Ma, Haiting Pan, Xiao Wang

**Affiliations:** a State Key Laboratory of Reproductive Regulation and Breeding of Grassland Livestock, School of Life Sciences, Inner Mongolia University, Hohhot, China; b Basic Medical College, Inner Mongolia Medical University, Hohhot, China; Wuhan Institute of Virology

**Keywords:** *Lactobacillus*, probiotic, *Staphylococcus aureus*, oral vaccine, mucosal delivery system

## Abstract

Staphylococcus aureus is a Gram-positive bacterium responsible for most hospital-acquired (nosocomial) and community-acquired infections worldwide. The only therapeutic strategy against S. aureus-induced infections, to date, is antibiotic treatment. A protective vaccine is urgently needed in view of the emergence of antibiotic-resistant strains associated with high-mortality cases; however, no such vaccine is currently available. In our previous work, the feasibility of implementing a *Lactobacillus* delivery system for development of S. aureus oral vaccine was first discussed. Here, we describe systematic screening and evaluation of protective effects of engineered *Lactobacillus* against S. aureus infection in terms of different delivery vehicle strains and S. aureus antigens and in localized and systemic infection models. Limosilactobacillus reuteri WXD171 was selected as the delivery vehicle strain based on its tolerance of the gastrointestinal environment, adhesion ability, and antimicrobial activities *in vitro* and *in vivo*. We designed, constructed, and evaluated engineered L. reuteri strains expressing various S. aureus antigens. Among these, engineered L. reuteri WXD171-IsdB displayed effective protection against S. aureus-induced localized infection (pneumonia and skin infection) and, furthermore, a substantial survival benefit in systemic infection (sepsis). WXD171-IsdB induced mucosal responses in gut-associated lymphoid tissues, as evidenced by increased production of secretory IgA and interleukin 17A (IL-17A) and proliferation of lymphocytes derived from Peyer’s patches. The probiotic L. reuteri-based oral vaccine appears to have strong potential as a prophylactic agent against S. aureus infections. Our findings regarding utilization of *Lactobacillus* delivery system in S. aureus vaccine development support the usefulness of this live vaccination strategy and its potential application in next-generation vaccine development.

**IMPORTANCE** We systematically screened and evaluated protective effects of engineered *Lactobacillus* against S. aureus infection in terms of differing delivery vehicle strains and S. aureus antigens and in localized and systemic infection models. Engineered L. reuteri was developed and showed strong protective effects against both types of S. aureus-induced infection. Our findings regarding the utilization of a *Lactobacillus* delivery system in S. aureus vaccine development support the usefulness of this live vaccination strategy and its potential application in next-generation vaccine development.

## INTRODUCTION

Staphylococcus aureus infection, the most common type of hospital-acquired (nosocomial) and community-acquired infection, is the cause of a variety of diseases ranging from mild soft tissue infection to fulminant sepsis or death (1). In humans and many other mammalian species, S. aureus colonizes various mucosal tissues. The anterior nares are the most frequent carriage site, and colonization there leads to a high risk of staphylococcal disease (2). S. aureus-induced skin and soft tissue infections (SSTI) and pneumonia are common localized infectious diseases (3). S. aureus is an opportunistic pathogen that generally causes infections at mucosal sites; however, it is sometimes able to pass from there to deeper tissues or the bloodstream (4). The presence of S. aureus in bloodstream (bacteremia) may lead to sepsis (a systemic inflammatory response to infection). An elevated risk of S. aureus bloodstream infection in patients undergoing general, orthopedic, or thoracic surgery, being treated in the intensive care unit, or undergoing hemodialysis or continuous peritoneal dialysis has been reported in many studies (1, 5). S. aureus-induced bloodstream infections are characterized by high mortality rates despite appropriate treatment (20 to 50%, depending on infection severity), frequent recurrence (5 to 10%), and lasting impairments in >33% of survivors (6).

The only viable treatment options for S. aureus-induced infectious diseases are antibiotics that directly target the pathogen. However, treatment efficacy is limited by the widespread occurrence of antibiotic-resistant and multidrug-resistant strains; a substantial percentage of patients experience persistent bacteremia or die from the disease despite aggressive therapy (7). In view of high morbidity and mortality in such cases, and inevitable increased exposure to antibiotic-resistant or multidrug-resistant strains, development of immune-based therapies for humans and farm animals is highly desirable (8). Unfortunately, all attempts so far to develop an effective vaccine against S. aureus have failed. Such failure, in clinical trials, may be related to a focus on single targets or development of humoral-based vaccines as opposed to vaccines based on a combination of antigens that induces both cellular and mucosal immunity (9). Sepsis often elicits a paradoxical immunosuppressive response that co-occurs with inflammation. Such a combination of inflammation and immunosuppression typically leads to collateral damage of local tissues, disruption of host defensive mechanisms against the causative pathogen, and secondary infections (10). Protective effects against invasive S. aureus infection have been reported for interleukin 17 (IL-17)-secreting T cells and Th1 memory cells (11). In many studies, a mucosal administration route has elicited both localized mucosal and disseminated systemic immune responses, through a mechanism termed the common mucosal immune system (12). Thus, mucosal delivery systems have potential application for protection against localized and systemic S. aureus infections.

Another potentially useful method is live probiotic vector-based delivery of antitoxin or therapeutic proteins, for targeted, low-cost release of these proteins in the intestinal tract (13). The genus *Lactobacillus* is a much-used model of live-delivery bacteria in view of several useful immunobiological features: anti-inflammatory and anticancer effects, control of immunity in autoimmune disease, immunogenic and immune-adjuvant properties, and tolerance of gastric acid and bile fluid (14, 15). Recombinant forms of various *Lactobacillus* species have been used as bases for vaccines against Streptococcus pneumoniae (16), transmissible gastroenteritis coronavirus (17), Bacillus anthracis (18), rotavirus (19), and tetanus toxin (20). *Lactobacillus*-based antigen delivery systems against localized S. aureus infections were demonstrated in our 2021 study (21); however, their possible application against systemic infections has not been investigated. The species generally known as Lactobacillus reuteri (renamed Limosilactobacillus reuteri in a 2020 taxonomic revision [22]) has been proposed as a novel probiotic in view of many host health-promoting properties, including modulation of immune responses, enhancement of mucosal integrity, inhibition of bacterial translocation, and secretion of antimicrobial metabolites (23). Studies to date of *Lactobacillus*-based delivery systems have focused on Lactobacillus plantarum (renamed Lactiplantibacillus plantarum in the 2020 revision) but have not examined the feasibility of using L. reuteri as a delivery vehicle (13).

The feasibility of implementing a *Lactobacillus* delivery system for development of an S. aureus oral vaccine was first discussed in our 2021 study (21). In the present follow-up study, the probiotic L. reuteri WXD171 was selected as a delivery vehicle strain, following systematic screening of six potentially useful strains, based on its tolerance of the gastrointestinal environment, adhesion ability, and antimicrobial activities *in vitro* and *in vivo*. Engineered L. reuteri was further developed to deliver five different S. aureus antigens which were identified as having potential in S. aureus vaccine research, and protective effects were evaluated not only in murine models of S. aureus-induced localized infection (pulmonary and skin infection) but also in systemic infection (sepsis). The probiotic *Lactobacillus*-based oral vaccine is a promising prophylactic agent against S. aureus infection. Our experimental findings provide a useful basis for future development of *Lactobacillus* delivery systems and of mucosal vaccines against S. aureus.

## RESULTS

### Probiotic properties of selected candidate delivery bacteria.

**FIG 1:**
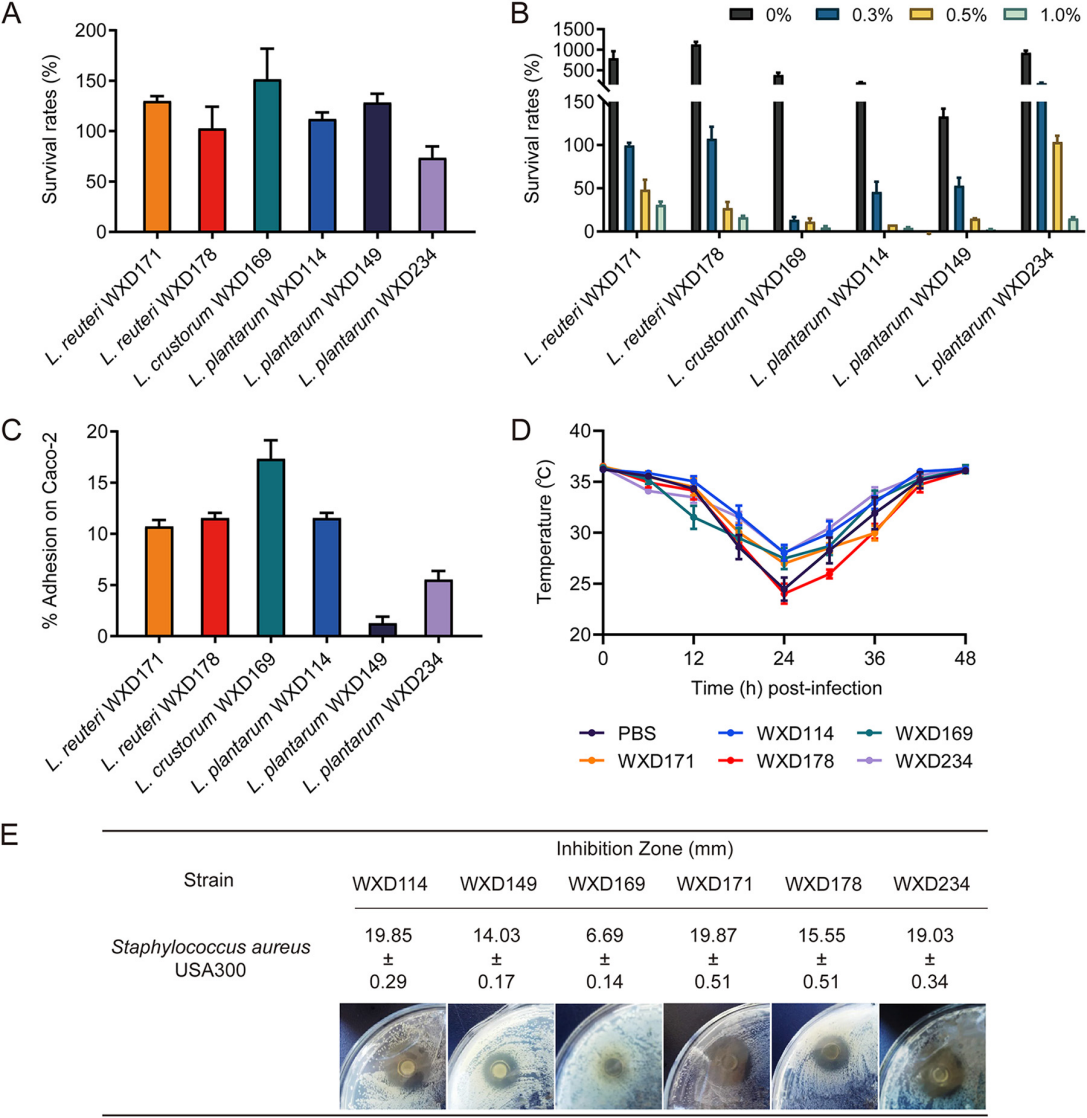
Probiotic properties of selected candidate delivery bacteria. (A) Survival rates of *Lactobacillus* strains cultured in the presence of simulated gastrointestinal fluid (*n* = 3). (B) Survival rates of strains in the presence of simulated intestinal fluid containing 0, 0.3, 0.5, or 1.0% bile salt (*n* = 3). (C) Percent adhesion of strains to Caco-2 cells (*n* = 3). (D) WXD171-IsdB-treated mice were inoculated via the left nostril with 40 μL bacterial slurry (5 × 10^9^ CFU of S. aureus USA300), and body temperatures were monitored for 48 h (*n* = 5). (E) *In vitro* antibacterial activity of six strains against S. aureus determined by the Oxford cup method (*n* = 3). Data are means and SD for each group.

*In vitro* screening was performed for survival of lactobacilli under simulated gastrointestinal conditions. Six *Lactobacillus* strains isolated from traditional dairy products were notably tolerant of these conditions: L. reuteri WXD171 and WXD178, Companilactobacillus crustorum (Lactobacillus crustorum) WXD169, and *L. plantarum* WXD114, WXD149, and WXD234. The effects of simulated gastrointestinal fluid and simulated intestinal fluid on survival rates of these strains are summarized in Fig. 1A and B. The adhesion ability of lactobacilli is considered an essential property of probiotics (24). The above strains (except WXD149) showed high or moderate adhesion (≥5%) to Caco-2 cells (Fig. 1C). WXD114, WXD171, WXD178, and WXD234 had significant inhibitory effects on *in vitro* S. aureus growth, with inhibition zone diameters of >15 mm in some cases (Fig. 1E). WXD114, WXD169, WXD171, and WXD234 ameliorated hypothermia symptoms postchallenge and displayed antibacterial activity *in vivo*, whereas such effects of WXD178 were negligible (Fig. 1D). In view of the combined results of the *in vitro* and *in vivo* experiments, we screened WXD171, WXD114, and WXD234, with probiotic properties, as candidate delivery bacteria. Previous studies of *Lactobacillus*-based delivery systems have focused on *L. plantarum* and have not addressed the feasibility of L. reuteri as a delivery vehicle (13, 25). L. reuteri present in gastrointestinal tracts of mammals displays a variety of probiotic properties and has been considered during the past decade as a novel probiotic bacterium (26). We therefore selected L. reuteri WXD171 as a delivery vehicle for development of a genetically engineered S. aureus oral vaccine.

### Cell surface display system for S. aureus antigen expression in transformed L. reuteri.

**FIG 2:**
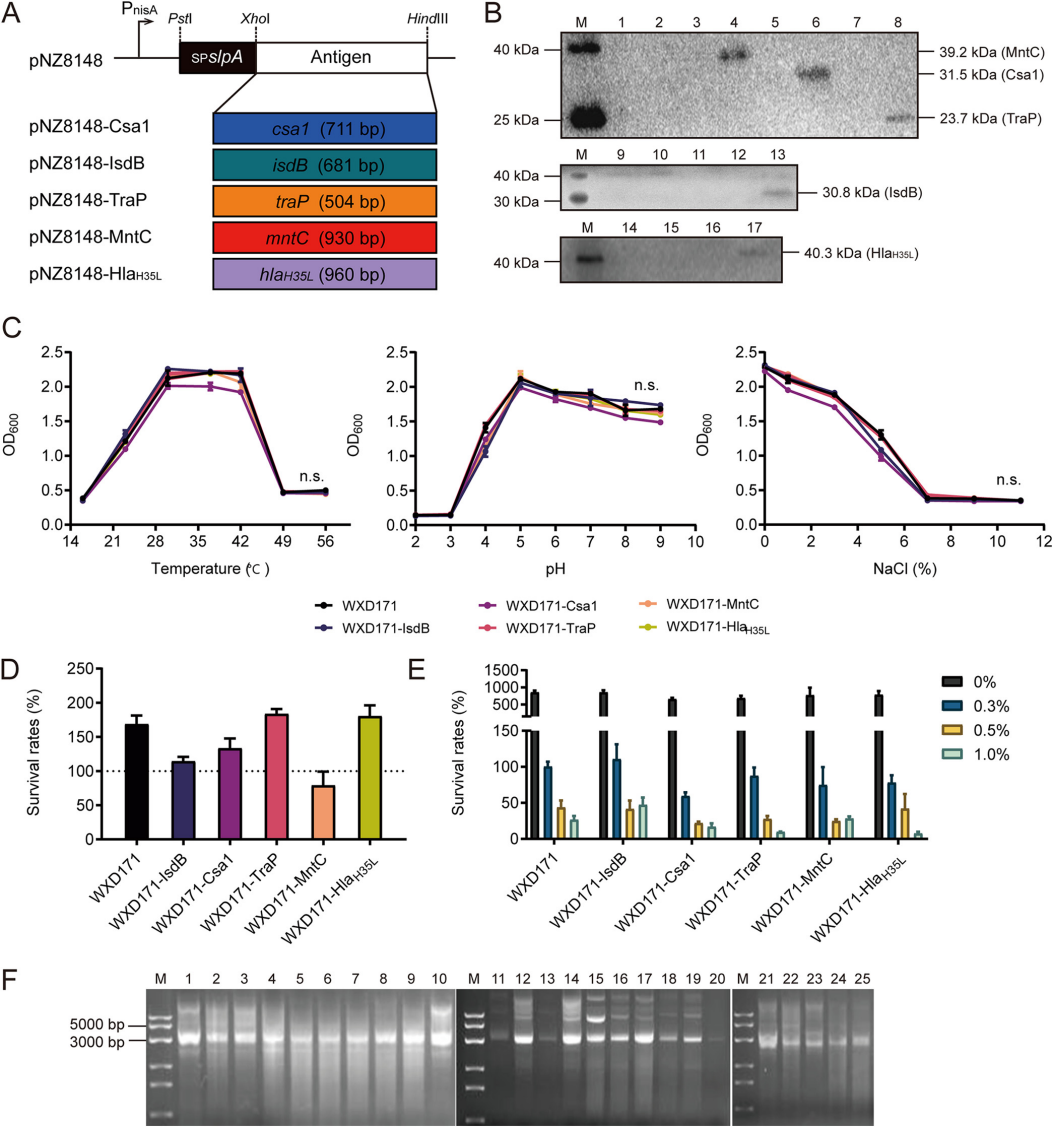
Construction and physiological characteristics of recombinant L. reuteri strains WXD171-Csa1, -IsdB, -TraP, -MntC, and -Hla_H35L_. (A) Genetic engineering using pNZ8148. Full-length coding sequences of Csa1, IsdB, TraP, MntC and Hla_H35L_ were cloned into pNZ8148 and modified with a signal peptide from L. brevis. (B) S. aureus antigen expression on recombinant L. reuteri strains subjected to nisin induction was analyzed by Western blotting. (Left) Molecular masses of prestained marker proteins. Lanes M, marker proteins; lanes 1 to 17, culture supernatants and cell extracts of WXD171-Ctrl (lanes 1, 9, 14; 2, 10, and 15), WXD171-MntC (lanes 3 and 4), WXD171-Csa1 (lanes 5 and 6), WXD171-TraP (lanes 7 and 8), WXD171-IsdB (lanes 12 and 13), and WXD171-Hla_H35L_ (lanes 16 and 17). (C) Growth status of strains at various temperatures, pH values, and NaCl contents (*n* = 3). (D) Survival rates of strains in the presence of simulated gastrointestinal fluid (*n* = 3). (E) Survival rates of strains in the presence of simulated intestinal fluid containing 0, 0.3, 0.5, or 1.0% bile salt (*n* = 3). (F) Extraction of recombinant plasmids pNZ8148-Csa1 (lanes 1 to 5), pNZ8148-IsdB (lanes 6 to 10), pNZ8148-Hla_H35L_ (lanes 11 to 15), pNZ8148-MntC (lanes 16 to 20), and pNZ8148-TraP (lanes 21 to 25) from strains at 5, 10, 15, 10, and 25 days. Data are means and SD for each group. n.s., not significant (comparison with the WXD171 group).

The feasibility of implementing a *Lactobacillus* delivery system for development of an S. aureus oral vaccine was discussed in our 2021 study (21). Five antigens utilized in this study are well-known staphylococcal virulence factors or structural proteins shown in previous studies to elicit protective immunity in animal models of staphylococcal infections. These antigens include conserved staphylococcal antigen (Csa1) (27), iron-regulated surface determinant protein B (IsdB) (28), target RNAIII-activating protein (Trap) (29), manganese transport protein C (MntC) (30), and α-hemolysin (Hla_H35L_) (31). In the present study, six genetically engineered strains based on L. reuteri were constructed for screening of strains with strong protective effects. A cell surface display system for S. aureus antigen expression in L. reuteri was designed using plasmid pNZ8148. The strategy for construction of plasmids pNZ8148-Csa1, -IsdB, -TraP, -MntC, and -Hla_H35L_ is shown schematically in Fig. 2A. Recombinant plasmids were constructed successfully and introduced into WXD171 by electroporation. Lysates of recombinant cells were analyzed by Western blotting, and specific signals were detected at molecular masses of 39.2 kDa (signal peptide [sp]-MntC), 31.5 kDa (sp-Csa1), 23.7 kDa (sp-TraP), 30.8 kDa (sp-IsdB), and 40.3 kDa (sp-Hla_H35L_), consistent with expected product sizes (Fig. 2B). S. aureus antigens were thus expressed successfully in WXD171. Recombinant L. reuteri and wild-type (WT) strains did not differ notably in terms of growth status at various temperatures, pH values, or NaCl contents (Fig. 2C). Recombinant strains retained tolerance of gastric acid (Fig. 2D) and bile salt (Fig. 2E), and recombinant plasmids showed stabilized inheritance until day 25 or more (Fig. 2F).

### Engineered L. reuteri had a strong protective effect against S. aureus-induced pulmonary infection.

**FIG 3:**
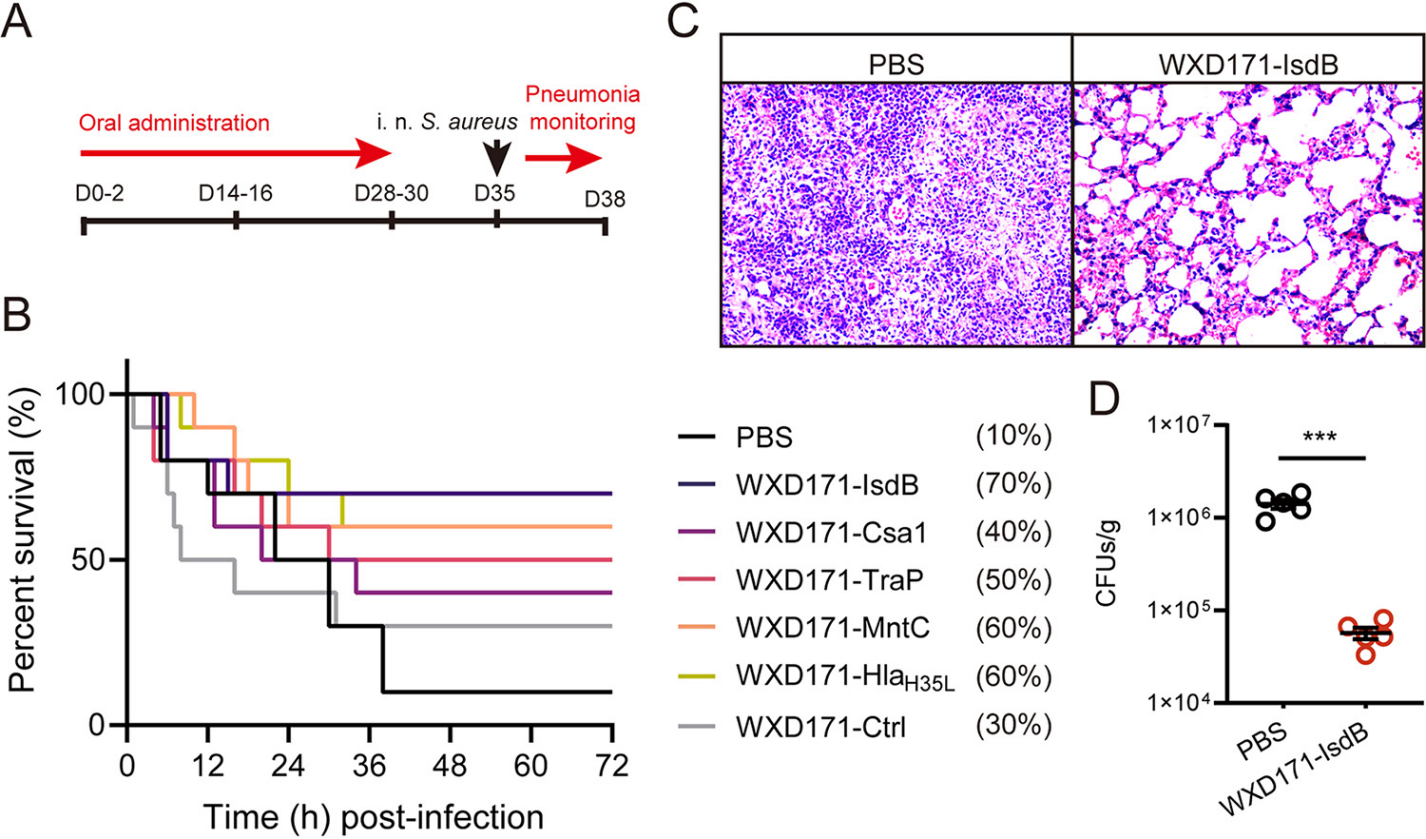
Engineered L. reuteri effectively protected against S. aureus-induced pulmonary infection. (A) WXD171-IsdB pretreatment in a mouse model of pulmonary infection (schematic). (B) WXD171-IsdB- and PBS-treated mice were challenged on day 35 with S. aureus USA300 (i.n. administration of 5 × 10^9^ CFU/40 μL/mouse) and held upright for 1 min. Survival rates were monitored for 72 h (*n* = 10). (C) WXD171-IsdB- and PBS-treated mice were challenged on day 35 with S. aureus USA300 (i.n. administration of 5 × 10^8^ CFU/40 μL/mouse) as described above, and histopathological evaluation of organ sections was performed by light microscopy (H&E staining; magnification, ×200). (D) Lung bacterial burdens (*n* = 5). ***, *P* < 0.001 (comparison with the PBS group). Data are means and SD for each group.

S. aureus is a leading cause of hospital-acquired (nosocomial) and community-acquired pneumonia, and these are the most common examples of S. aureus-induced localized infection (32). We evaluated the protective effects of six recombinant L. reuteri strains (see “Probiotic properties of selected candidate delivery bacteria”) in terms of resistance to S. aureus localized infection in our mouse model. Survival rates following intranasal challenge were much higher for WXD171-IsdB (70%), WXD171-Csa1 (40%), WXD171-TraP (50%), WXD171-MntC (60%), and WXD171-Hla_H35L_ (60%) groups than for the control (phosphate-buffered saline [PBS]) group (10%) (Fig. 3B). Histological features and lung bacterial burden were evaluated for the WXD171-IsdB group, which had the highest survival rate. WXD171-IsdB had low degrees of lung tissue damage and inflammation, whereas the PBS group showed severe histopathological damage, including alveolar wall fragmentation, lymphocyte infiltration, and a high degree of erythrocyte exudation (Fig. 3C). Lung bacterial burden was significantly lower for the WXD171 group than for the PBS group (Fig. 3D). These findings indicate that recombinant strain WXD171-IsdB had a strong protective effect against S. aureus-induced pneumonia and ameliorated histopathological damage and that probiotic-based immunotherapy may effectively protect against S. aureus-induced pulmonary infection.

### Engineered L. reuteri had a protective effect against S. aureus-induced skin infection.

**FIG 4:**
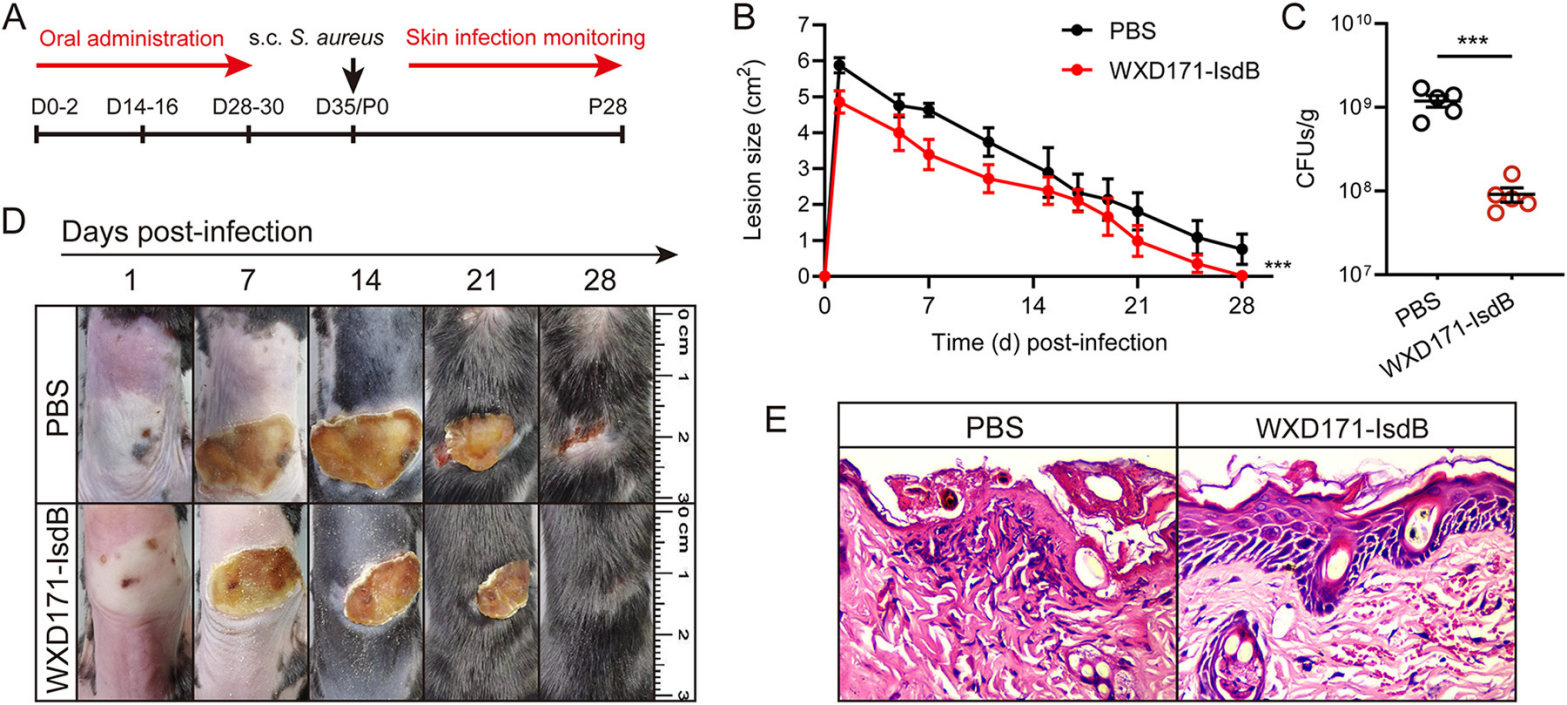
Engineered L. reuteri effectively protected against S. aureus-induced skin infection. (A) WXD171-IsdB pretreatment in a mouse model of skin infection. (B) WXD171-IsdB- and PBS-treated mice were challenged on day 35 with S. aureus USA300 (dorsal s.c. injection of 5 × 10^8^ CFU/50 μL/mouse), and lesion size was monitored for 28 days (*n* = 5). Mean lesion sizes ± SEM (*n* = 5) are shown. (C) Numbers of bacteria in lesions (*n* = 5) at 48 h postinfection. (D) Representative photographs of skin lesions. (E) Histopathological evaluation of organ sections by light microscopy (H&E staining; magnification, ×400). Data are means and SD for each group. ***, *P* < 0.001 (comparison with the PBS group).

S. aureus is responsible for ~76% of skin and soft tissue infections in humans (33), despite the fact that its skin colonization rate (34) and abundance levels (35) are much lower than those of other bacterial skin colonizers. We constructed an S. aureus-induced skin infection model to evaluate the protective effect of WXD171-IsdB. Mice were inoculated intradermally with S. aureus USA300, which led to development of visible skin lesions. Lesions in the WXD171-IsdB group reached diameters up to 4.86 ± 0.31 cm^2^ by day 1 postinfection and healed by day 28, whereas lesions in the PBS group were much larger (5.88 ± 0.22 cm^2^) by day 1 and not completely healed by day 28 (Fig. 4B and D). On day 2, bacterial burden at the infection site was significantly lower for the WXD171-IsdB group than for the PBS group (Fig. 4C). The WXD171-IsdB group had minor tissue damage and inflammation at the infection site, whereas the PBS group showed severe histopathological damage, including neutrophil infiltration and epidermal thickening (Fig. 4E). These findings indicate that WXD171-IsdB had a protective effect against S. aureus-induced skin infection.

### Engineered L. reuteri had a protective effect against S. aureus-induced systemic infection.

**FIG 5:**
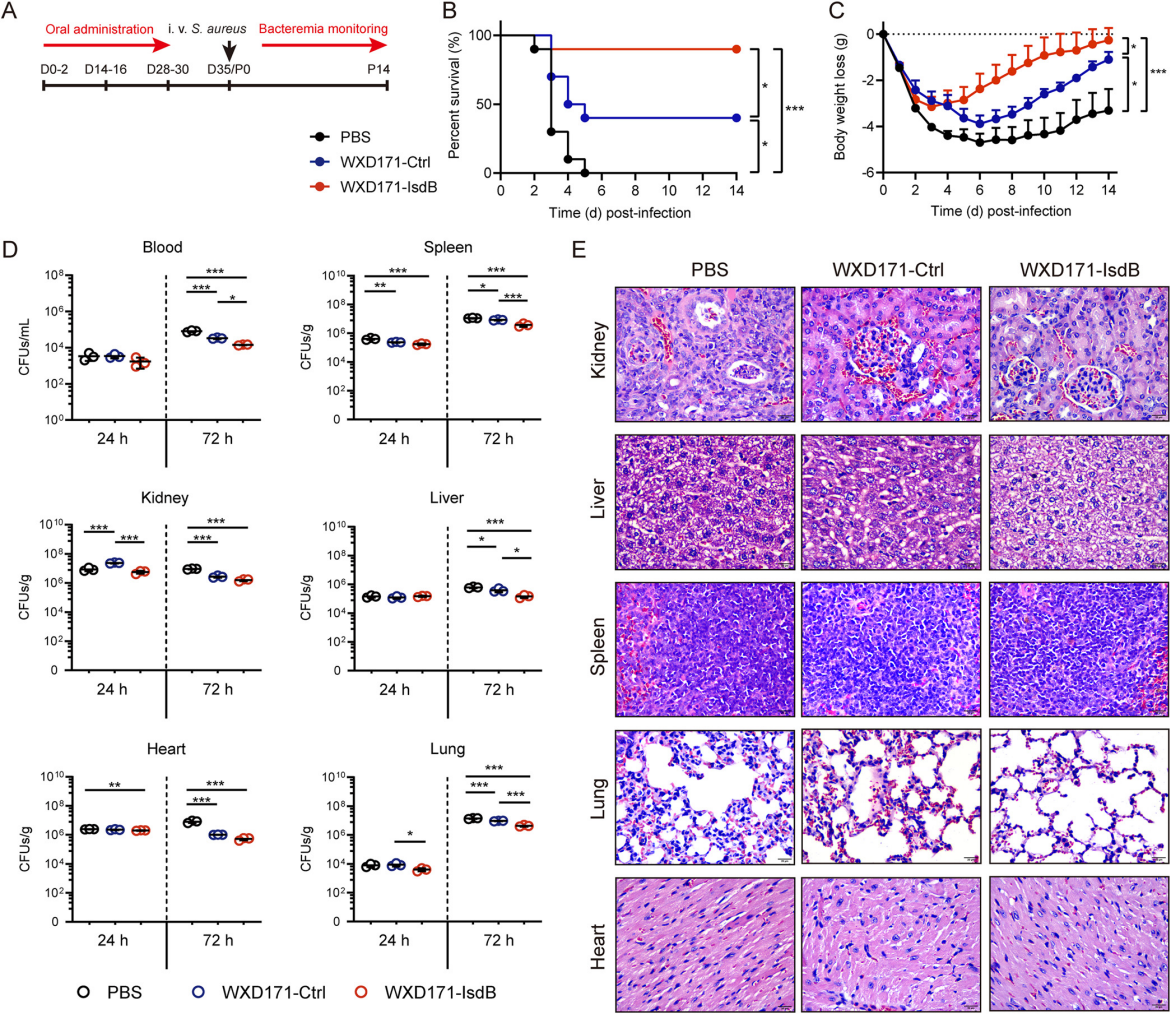
Engineered L. reuteri effectively protected against S. aureus-induced systemic infection. (A) WXD171-IsdB pretreatment in a mouse model of blood infection. (B) WXD171-IsdB- and PBS-treated mice were challenged on day 35 with S. aureus USA300 (i.v. administration of 1 × 10^7^ CFU/100 μL/mouse), and survival rates were monitored for 8 days (*n* = 10). (C) WXD171-IsdB- and PBS-treated mice were challenged on day 35 with S. aureus USA300 (i.v. administration of 2 × 10^6^ CFU/100 μL/mouse) as described above. The body weight loss timeline (*n* = 10) is shown. (D) Effect of WXD171-IsdB on S. aureus burden during systemic infection (*n* = 3). (E) Histopathological evaluation of organ sections by light microscopy (H&E staining; magnification, ×200). Data are means and SD for each group. *, *P* < 0.05; **, *P* < 0.01; ***, *P* < 0.001 (comparison with the PBS group).

Strong protective effects of probiotic-based immunotherapy in models of localized S. aureus-induced infection were observed in the above-described experiments. We further investigated the protective effects of WXD171-IsdB in models of S. aureus-induced systemic infection. Following intravenous (i.v.) injection of lethal doses of S. aureus USA300, the survival rate was much higher for the group receiving WXD171-IsdB (90%; 9/10) than for the group receiving antigen-free WXD171-Ctrl (40%; 4/10). Mice in the PBS group were all dead by day 5 (0%; 0/10) (Fig. 5B). Following injection of sublethal-dose S. aureus USA300, the degree of weight loss was less for the WXD171-IsdB group than for the WXD171-Ctrl and PBS groups (Fig. 5C). For the WXD171-IsdB group, weight dropped to a minimal value (weight loss, ~3.2 g), then began increasing, and by day 14 was close to the initial value. For the PBS group, weight loss was more severe and prolonged; weight dropped to a minimal value (weight loss, ~4.7 g), then increased slowly, and by day 14 was still significantly lower than the initial value (weight loss, ~3.3 g). Weight loss in the WXD171-Ctrl group was ameliorated to some degree, but much less strongly than for the WXD171-IsdB group (Fig. 5C). In the WXD171-IsdB group, bacterial burden on days 1 and 3 was significantly lower than in the PBS group; on day 3, it was reduced ~3-fold in the spleen and lungs, 4-fold in the liver, and 6-fold in the blood and kidneys (Fig. 5D). The degree of histopathological damage was variable for most organs (Fig. 5E). The WXD171-IsdB group showed mild inflammatory responses, whereas the PBS group had severe histopathological damage, including lymphocyte infiltration and a high degree of erythrocyte exudation. The degree of protective effect was moderate for WXD171-Ctrl, i.e., much less than for WXD171-IsdB. The *Lactobacillus* vector and IsdB both contributed to this effect. The above findings, taken together, clearly demonstrate therapeutic effects of WXD171-IsdB (promotion of survival and reduction of weight loss, inflammation, and bacterial burden) and its potential application for protection against S. aureus-induced systemic infection.

### Engineered L. reuteri elicited mucus sIgA- and serum IgG-mediated immune responses.

**FIG 6:**
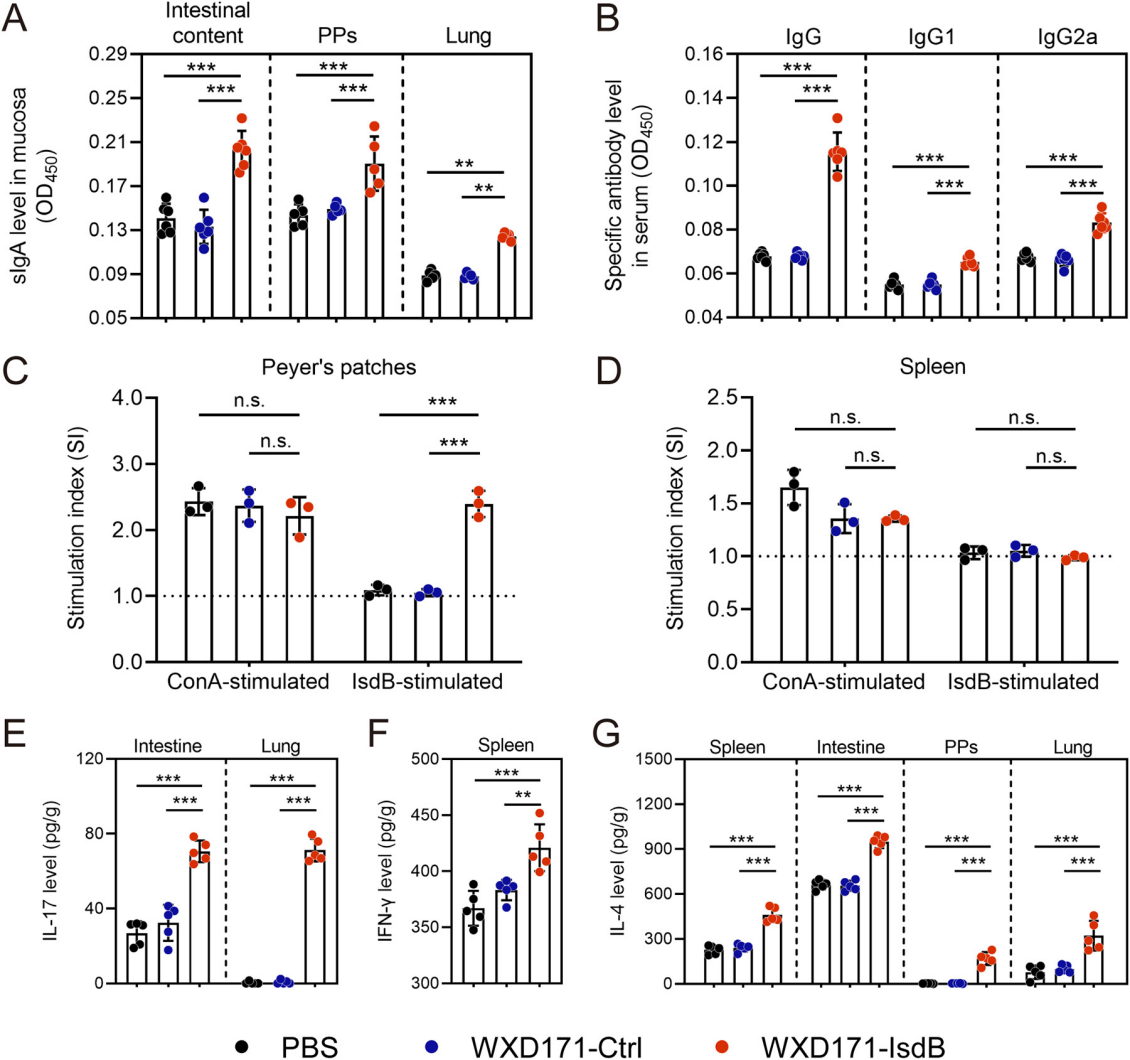
Characteristics of immune cell responses induced by WXD171-IsdB treatment. (A and B) Mice were treated with WXD171-IsdB, WXD171-Ctrl, or PBS; samples were collected on day 35, and sIgA and IgG levels were measured. (A) Mean sIgA levels at mucosal sites (*n* = 5). (B) Mean IsdB-specific IgG levels in serum (*n* = 5). (C and D) T cells from PPs and spleens were prepared and restimulated with IsdB or ConA protein, and an MTS assay of antigen-specific T cells was performed (*n* = 3). (C) Mean T cell SI for PPs (*n* = 3). (D) Mean T cell SI for spleen (*n* = 3). (E to G) WXD171-IsdB-induced IL-17, IFN-γ, and IL-4 production in spleen and mucosal sites was determined by ELISA. (E) Mean IL-17 levels in intestinal tissue and lung (*n* = 5). (F) Mean IFN-γ levels for spleen (*n* = 5). (G) Mean IL-4 levels for spleen, intestinal tissue, PPs, and lung (*n* = 5). Data are means and SD for each group. **, *P* < 0.01; ***, *P* < 0.001; n.s., not significant (comparison with the PBS group).

The mechanism whereby WXD171-IsdB protects against S. aureus-induced systemic infection was investigated by measuring levels of serum IgG and mucus secretory IgA (sIgA), which reflect activation of humoral and mucosal immunity from an antibody perspective. sIgA levels assayed on day 35 at mucosal sites (intestinal contents, Peyer’s patches [PPs], lungs), and IsdB-specific IgG serum levels, were both significantly higher for WXD171-IsdB than for the WXD171-Ctrl and PBS groups (Fig. 6A and B). These findings indicate that WXD171-IsdB treatment elicited mucus sIgA- and serum IgG-mediated immune responses.

### Engineered L. reuteri enhanced lymphocyte proliferation in PPs.

The effect of WXD171-IsdB on cell-mediated immunity was evaluated by preparing lymphocyte suspensions from PPs and spleen on day 35 and performing an MTS [3-(4,5-dimethylthiazol-2-yl)-5-(3-carboxymethoxyphenyl)-2-(4-sulfophenyl)-2H-tetrazolium] assay of lymphocyte proliferation (see “ELISA for determination of IgG and sIgA levels”). Proliferation of both IsdB- and ConA-stimulated PP lymphocytes was enhanced strongly in the WXD171-IsdB group but weakly in the WXD171-Ctrl and PBS groups (Fig. 6C). These findings suggest that WXD171-IsdB stimulated cell-mediated immune responses in gut-associated lymphoid tissues.

### Engineered L. reuteri enhanced production of IL-4, IFN-γ, and IL-17 in tissues.

T-helper (Th) cells, which differentiate into Th1, Th2, and Th17 subsets (36), play essential roles in promoting immune responses following bacterial infection or immunization (37–39). Cytokines secreted by these cells are categorized as Th1-related (gamma interferon [IFN-γ]), Th2-related (IL-4), and Th17-related (IL-17) cytokines (40, 41). Th1-mediated and Th17-mediated immunity are associated with protection from S. aureus infection (42–44). For investigation of WXD171-IsdB effects on production of IL-17, IFN-γ, and IL-4, we prepared supernatants of intestinal contents, PPs, and lung and spleen homogenates. Levels of IL-17 in intestinal tissue (Fig. 6E), IFN-γ in spleen (Fig. 6F), and IL-4 in intestinal tissue and spleen (Fig. 6G) were all significantly higher in the WXD171-IsdB group than other groups. These findings demonstrate induction of Th1-, Th2-, and Th17-mediated immune responses by WXD171-IsdB.

### Engineered L. reuteri induced immune cell responses.

**FIG 7:**
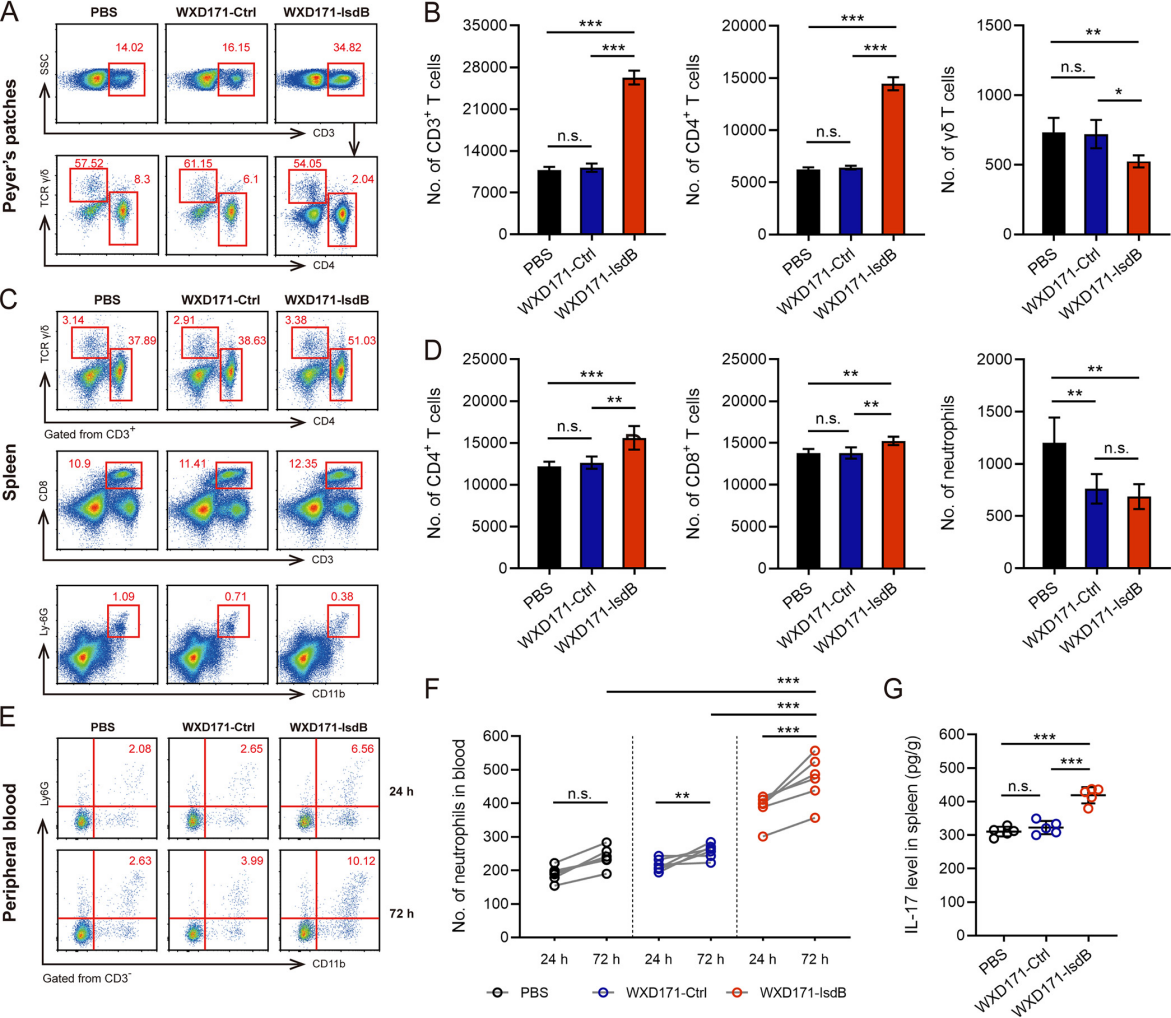
WXD171-IsdB treatment increased numbers of CD3^+^, CD4^+^, and CD8^+^ cells and recruited neutrophils to peripheral blood postinfection. (A) Representative histograms of frequencies of live CD3^+^, CD3^+^ CD4^+^, and CD3^+^ TCRγ/δ^+^ cells in PPs on day 35. (B) Population count statistics (*n* = 5) for three types of cells. (C) Representative histograms of frequencies of live CD4^+^, CD8^+^, and CD11b^+^ Ly6G^+^ cells in spleens on day 35. (D) Distinct cell population count statistics (*n* = 5). (E) Representative histograms of frequencies of neutrophils in peripheral blood at 24 or 72 h postinfection. (F) Population count statistics (*n* = 5) for three types of cells. (G) Mean IL-17 levels in spleens at 72 h postinfection (*n* = 5). Data are means and SD for each group. *, *P* < 0.05; **, *P* < 0.01; ***, *P* < 0.001; n.s., not significant (comparison with the PBS group).

We further investigated the protective mechanism of WXD171-IsdB by analyzing responses of immune cells in PPs and spleen to probiotic stimulation. PPs are composed mainly of B cells and T cells (particularly CD4^+^ T cells) and are the primary site of immune responses to immunomodulators (45). γδ T cells, located in the intestinal barrier, function as intermediaries between innate and adaptive immune responses (46). We performed flow-cytometric assays of CD3^+^, CD4^+^, and T-cell receptor γ/δ-positive (TCRγ/δ^+^) cell populations. Numbers of CD3^+^ and CD4^+^ (but not γδ^+^) T cells were significantly higher in PPs from the WXD171-IsdB group than other groups (Fig. 7A and B). This finding indicates that WXD171-IsdB elicited immune response by activating CD4^+^ T cells. Probiotic bacteria have been suggested to play a role in regulation of γδ T cells; however, orally administered WXD171-IsdB had no notable effect on these cells. Uptake of bacteria from the bloodstream occurs mainly in the spleen (47). Accordingly, we examined responses to probiotic treatment of CD4^+^ T cells, CD8^+^ T cells, and neutrophils in spleen. Numbers of CD4^+^ and CD8^+^ T cells showed large increases, whereas that of neutrophils declined (Fig. 7C and D). These findings suggest that WXD171-IsdB treatment, in addition to stimulating localized immune response in intestinal mucosa, elicited a systemic immune response that blocked S. aureus invasion.

### Engineered L. reuteri increased the number of circulating neutrophils in the bloodstream following infection.

Th cells help coordinate response of phagocytic effector cells to clear infection but do not directly target bacteria or infected cells. Macrophages and cytotoxic T cells are activated by IFN-γ, while IL-17 recruits neutrophils to infection sites (48). A notable increase of IL-17 and IFN-γ levels by WXD171-IsdB treatment was observed in our 2021 study. Spleen IL-17 and IFN-γ levels were measured 3 days postinfection in the present study. The WXD171-IsdB group, relative to other groups, showed a significantly higher level of IL-17 (Fig. 7G) but not of IFN-γ (data not shown). A flow-cytometric assay of circulating neutrophils in bloodstream was performed 24 and 72 h postinfection. Neutrophil numbers were higher in the WXD171-IsdB group than in other groups at 24 h and even more so at 72 h (Fig. 7E and F). Bloodstream neutrophils recruited at the infection site through chemotactic stimuli have been shown to act as a second line of phagocytic defense, which promotes clearance of S. aureus (1, 47, 49). Our findings indicate that WXD171-IsdB treatment exerts its immunoprotective effect by increasing the number of circulating neutrophils.

## DISCUSSION

The incidence and disease severity of S. aureus infections have increased considerably worldwide during the past 2 decades, largely because of emergence of hypervirulent and multidrug-resistant strains (50). Standard antibiotic treatments currently prescribed for diseases caused by antibiotic-resistant S. aureus have various undesirable side effects, including occasional induction of dysbiosis of commensal bacteria, leading to increased susceptibility to other pathogens (51, 52). Attempts by large international drug companies (Merck, Pfizer, Novartis, and GSK) to develop vaccines against S. aureus have so far been unsuccessful (44). Factors that may contribute to such failures include (i) the lack of an adjuvant or delivery system for use in vaccines and (ii) a focus on generating high titers of antibodies against S. aureus surface antigens to facilitate antibody-mediated bacterial clearance, with disregard for the importance of cellular (particularly mucosal) immune responses (53).

Members of the genus *Lactobacillus* are useful vehicles because of their immunoadjuvant activities and delivery properties. The ability of L. reuteri WXD171 to tolerate the gastrointestinal environment demonstrated in this study allowed the carriage antigen to remain in the intestine for a long time and promoted antigen presentation and production of protective specific antibody (Fig. 1). *Lactobacillus* is safer than attenuated bacterial vectors because of a relative lack of potential risk of pathogenicity recurrence, side effects of varying severity, or immunogenicity/reactogenicity imbalance. *Lactobacillus*, as an adjuvant, affects innate immune cells such as dendritic cells (DCs), which promote antigen presentation and production of protective antigen-specific antibodies in response to vaccination (54). *Lactobacillus* was shown to activate DCs to stimulate immune cells to release IL-17A and sIgA at the mucosal surface (52, 55). Previous studies have demonstrated important roles of sIgA in S. aureus clearance and of IL-17A in activation of polymorphonuclear neutrophils (PMN) to enhance their cytotoxicity against S. aureus (56, 57).

Different species of *Lactobacillus*, even different strains, show diverse adjuvant properties against pathogen infections (58). Both vehicle bacteria and the antigen contribute to the protective efficacy provided by a bacterial vaccine. The feasibility of implementing a *Lactobacillus* delivery system for development of an S. aureus oral vaccine was first discussed in our 2021 report; however, that study lacked evaluation/screening of alternative *Lactobacillus* strains or other S. aureus antigens. In the present study, we systematically evaluated/screened six *Lactobacillus* strains as potential delivery vehicles in terms of tolerance of the gastrointestinal environment, adhesion ability, and antimicrobial activity *in vitro* and *in vivo*. Further development and evaluation of engineered strains in combination with various antigens led to the selection of the strain with the strongest protective effect.

S. aureus is an opportunistic pathogen that generally causes infections at mucosal sites. However, in certain cases, particularly in patients who are elderly, are immunodeficient, or have undergone cardiac surgery, it can penetrate the mucosal barrier and enter the bloodstream or deeper tissues, leading to bacteremia or even sepsis (1, 59). Numerous studies have focused on protective effects of nanoparticle-based S. aureus mucosal vaccines against localized infections (60), but very few have addressed the possible utilization of such an approach against systemic infections. Our 2021 study described the *Lactobacillus* S-layer display system as a construction strategy and Hla_H35L_ as a proof-of-concept antigen for the design of an oral vaccine targeting localized infections. In the present study, this construction strategy was used to produce engineered L. reuteri expressing five well-known antigens, including staphylococcal virulence factors and structural proteins, and their protective effects were evaluated in mouse models of localized and systemic infection. This is the first demonstration of a protective effect of engineered L. reuteri against S. aureus-induced pneumonia, skin infection, and sepsis, which are representative of the range of S. aureus infections in humans.

S. aureus typically occupies mucosal niches and invades host tissues via mucosal surface (61). Prevention of infection by formation of a strong mucosal barrier at the first point of contact with host tissue is an effective strategy. The central role of sIgA in protection of mucosal tissues against pathogens is evidenced by its ability to block attachment to surface epithelium of pathogens or their toxic products (62). Oral immunization with engineered L. reuteri triggered a local sIgA immune response in intestinal mucosa and also enhanced sIgA levels at distant mucosal sites in the lung (Fig. 6A), thereby strengthening host immune defense against S. aureus-induced pneumonia. Oral immunization also increased serum-specific IgG levels. These findings indicate that activation of immune responses by oral delivery with engineered *Lactobacillus* was not limited to local sites.

Aberrant development of Th1 and Th17 cells in humans is associated with increased incidence of S. aureus infections, suggesting that these cells play key roles in mouse models of defense against S. aureus infections. In our 2021 study, IL-17A-deficient mice showed greatly reduced protective effects of engineered *Lactobacillus* vaccines. Th cells play major roles in controlling anti-infection responses of phagocytic effector cells: Th1 cells produce IFN-γ, which activates macrophages and cytotoxic T cells, while Th17 cells produce IL-17A, which recruits neutrophils to infection sites. In the present study, IFN-γ and IL-17A levels were higher in the group receiving engineered L. reuteri WXD171-IsdB than in other groups, demonstrating the ability of this strain to induce an effective immune response against S. aureus. Similarly, a mucosal-route S. aureus nanoemulsion vaccine in previous studies induced strong mucosal and systemic immune responses with high sIgA, IL-17A, and IFN-γ levels and displayed strong protective effects in sepsis and pneumonia models (60, 63). Boyaka observed that proinflammatory cytokines (IFN-γ and IL-17A) enhanced polymeric Ig receptor (pIgR) expression and thereby promoted production of high-affinity sIgA by epithelial cells (62). IL-17 helps control granulocytosis and neutrophil recruitment during bacterial infection, and IL-17 knockout mice showed reduced neutrophil levels associated with impaired host immune defenses and increased susceptibility to bacterial sepsis (64). Immunization with engineered L. reuteri in the present study increased numbers of circulating neutrophils in the bloodstream following infection (Fig. 7E), thereby enhancing host defense against S. aureus sepsis. In this study, immunization with engineered L. reuteri increased proliferation of PP lymphocytes, but not of spleen lymphocytes, in response to antigen stimulation (Fig. 6C, D). One possibility is that orally administered L. reuteri WXD171-IsdB activates only gut-associated lymphoid tissues, since the antigen does not reach the bloodstream; however, this concept remains to be investigated. The above findings, taken together, provide a preliminary explanation for the protective effects of engineered L. reuteri in our mouse model of S. aureus infection. The molecular mechanism of this effect is unclear and is the subject of ongoing studies.

In summary, we systematically screened and evaluated protective effects of engineered *Lactobacillus* against S. aureus infection in terms of differing delivery vehicle strains and S. aureus antigens and in localized and systemic infection models. The engineered L. reuteri strain WXD171-IsdB showed strong protective effects against both types of S. aureus-induced infection. Our findings regarding the utilization of a *Lactobacillus* delivery system in S. aureus vaccine development support the usefulness of this live vaccination strategy and its potential application in next-generation vaccine development.

## MATERIALS AND METHODS

### Bacterial strains and growth conditions.

Strains and plasmids used are listed in Table 1. *Lactobacillus* strains were grown in de Man, Rogosa, and Sharpe (MRS) broth (HuanKai Microbial Sci. & Tech., Guangdong, China) for 24 h at 37°C under anaerobic conditions. The *Lactobacillus* derivatives were cultured with the addition of chloramphenicol (20 μg/mL; antibiotic) and nisin (5 ng/mL; inducer). S. aureus USA300 was cultured in Luria-Bertani (LB) medium for 16 h at 37°C under aerobic conditions.

**TABLE 1:** Strains and plasmids used in this study

Strain or plasmid	Description	Source or reference
Strains		
WXD114	WT *Lactiplantibacillus plantarum*	Isolated from dairy products
WXD149	WT *Lactiplantibacillus plantarum*	Isolated from dairy products
WXD169	WT Companilactobacillus crustorum	Isolated from dairy products
WXD171	WT Limosilactobacillus reuteri	Isolated from dairy products
WXD178	WT Limosilactobacillus reuteri	Isolated from dairy products
WXD234	WT *Lactiplantibacillus plantarum*	Isolated from dairy products
WXD171-Ctrl	Derivative of WXD171 carrying pNZ8148	This study
WXD171-IsdB	Derivative of WXD171 carrying pNZ8148-IsdB	This study
WXD171-Csa1	Derivative of WXD171 carrying pNZ8148-Csa1	This study
WXD171-Hla_H35L_	Derivative of WXD171 carrying pNZ8148- Hla_H35L_	This study
WXD171-MntC	Derivative of WXD171 carrying pNZ8148-MntC	This study
WXD171-TraP	Derivative of WXD171 carrying pNZ8148-TraP	This study
USA300	S. aureus USA300	American Type Culture Collection (Manassas, VA, USA)
Plasmids		
pUC57-IsdB	Amp^r^; pUC57::_SP_*slpA*::His tag::*isdB*	GenScript Bio Co.
pUC57-Csa1	Amp^r^; pUC57::_SP_*slpA*::His tag::*csa1*	GenScript Bio Co.
pUC57-Hla_H35L_	Amp^r^; pUC57::_SP_*slpA*::His tag::*hla_H35L_*	GenScript Bio Co.
pUC57-MntC	Amp^r^; pUC57::_SP_*slpA*::His tag::*mntC*	GenScript Bio Co.
pUC57-TraP	Amp^r^; pUC57::_SP_*slpA*::His tag::*traP*	GenScript Bio Co.
pNZ8148	Cm^r^; L. lactis expression vector; *P_nisA_* promoter	This study (69)
pNZ8148-IsdB	Cm^r^; pNZ8148::_SP_*slpA*::His tag::*isdB*	This study
pNZ8148-Csa1	Cm^r^; pNZ8148::_SP_*slpA*::His tag::*csa1*	This study
pNZ8148-Hla_H35L_	Cm^r^; pNZ8148::_SP_*slpA*::His tag::*hla_H35L_*	This study
pNZ8148-MntC	Cm^r^; pNZ8148::_SP_*slpA*::His tag::*mntC*	This study
pNZ8148-TraP	Cm^r^; pNZ8148::_SP_*slpA*::His tag::*traP*	This study

### Experimental animals.

Specific-pathogen-free (SPF) female wild-type (WT) C57BL/6 mice, 6 to 8 weeks old, were from Beijing Vital River Laboratory Animal Technology Co. Experimental animal protocols were performed in accordance with guidelines of the Ethics Committee of Inner Mongolia University (IMU-mouse-2020-038).

### Screening of probiotic *Lactobacillus* for use as a delivery vehicle.

In our previous study, 73 *Lactobacillus* strains were isolated and purified from traditional dairy products in Inner Mongolia and identified on the basis of physiological characteristics and 16S rRNA gene sequence analysis (data not shown). In the present study, probiotic strains were screened with regard to gastrointestinal environmental tolerance, adhesion ability, and inhibitory properties.

For evaluation of acid tolerance, overnight cultures of candidate strains were washed and resuspended in simulated gastrointestinal fluid (0.2% NaCl and 0.35% pepsin dissolved in distilled water, adjusted to pH 3.0, and filter [pore size, 0.22 μm] sterilized) for 3 h at 37°C. Survival rates were calculated as (viable colony count at 3 h/viable colony count at 0 h) × 100. For evaluation of bile tolerance, overnight cultures were inoculated (1:25) in simulated intestinal fluid (MRS broth with 0.68% KH_2_PO_4_ and 1% trypsin; bile salt content, 0%, 0.3%, 0.5%, or 1.0%) for 12 h at 37°C. Survival rates were calculated as (viable colony count at 12 h/viable colony count at 0 h) × 100.

For evaluation of adhesion ability, strains were cultured overnight, washed 3× with PBS, resuspended in Dulbecco’s modified Eagle medium (DMEM) containing 10% fetal calf serum (FCS), and transferred onto Caco-2 monolayers (cultured 15 days) at a multiplicity of exposure (MOE) of ~10, and plates were incubated for 90 min at 37°C in a 5% CO_2_ atmosphere. Cell monolayers were rinsed with DMEM to remove unbound bacteria and lysed with 0.1% Triton X-100 in PBS to release adherent bacteria, and CFU of adherent bacteria were counted by plating serial dilutions of lysates on MRS agar plates. The percent adherent bacteria was calculated as (number of viable bacteria recovered on the basal side/number of viable bacteria added on the apical side) × 100 (65).

Inhibition of S. aureus growth by *Lactobacillus* strains was evaluated by the Oxford cup method. In brief, S. aureus USA300 was cultured overnight and diluted with LB medium to 10^8^ CFU/mL, and 100 μL diluted culture was use to evenly coat an LB agar plate. A sterile Oxford cup was placed on the plate, 100 μL *Lactobacillus* culture solution was added, and the plate was incubated for 24 h at 37°C. Inhibitory activity was quantified based on mean diameters of transparent circles around the cup. High inhibitory activity was defined as an inhibition halo of >15 mm.

*In vivo* antibacterial activity of orally administered *Lactobacillus* strains was assayed in a mouse model of S. aureus-induced pulmonary infection (see “Immunization”). Freshly grown *Lactobacillus* strains (1 × 10^9^ CFU/200 μL/mouse) were administered daily for 14 days, and then mice were intranasally (i.n.) inoculated with S. aureus USA300 (5 × 10^8^ CFU/40 μL/mouse). Body temperature was recorded every 6 h.

### Construction of a cell surface display system for S. aureus antigen expression based on L. reuteri WXD171.

Cell surface display recombinant plasmids pNZ8148-Csa1, -IsdB, -Trap, -MntC, and -Hla_H35L_ were constructed as described in our 2021 study, with the design strategy shown schematically in Fig. 2A. A 90-bp fragment encoding the Levilactobacillus brevis S-layer 30-amino-acid signal peptide (21) was ligated to the front of the S. aureus antigen. *csa1*, *isdB*, *traP*, *mntC*, and *hla_H35L_* genes were designed based on L. reuteri codon bias and synthesized artificially by GenScript Biotech (Nanjing). Fragments obtained from cloning plasmids pUC57-Csa1, -IsdB, -TraP, -MntC, and -Hla_H35L_ by digestion with PstI and HindIII were inserted into the Lactococcus lactis-inducible expression plasmid pNZ8148. Competent L. reuteri was prepared through inoculated (1:25) WXD171 in MRS containing 1% glycine. WXD171 were cultured until reaching a range of optical density at 600 nm (OD_600_) of 0.6 to 0.8, collected by centrifugation, washed twice, and suspended in 10% glycerol. Recombinant plasmids pNZ8148-Csa1, -IsdB, -TraP, -MntC, and -Hla_H35L_ were then introduced immediately into competent WXD171 by electroporation. Clones with insertion of the target gene were screened based on identification of antibiotic-resistant *Lactobacillus* by enzyme restriction, PCR, and sequencing. Positive clones of WXD171-Csa1, -IsdB, -TraP, -MntC, and -Hla_H35L_ were frozen (−80°C) in MRS containing 15% glycerol.

S. aureus antigen expression in WXD171 was analyzed by Western blotting. WXD171-Csa1, -IsdB, -TraP, -MntC, and -Hla_H35L_ were cultured in MRS at 37°C until an OD_600_ range of 0.6 to 0.8 was reached, induced with 5 ng/mL nisin (Sigma-Aldrich, St. Louis, MO, USA), and cultured overnight. Cells were washed twice with 0.01 M PBS (Coolaber Science & Technology Co., Beijing, China), lysed in PBS containing 10 mg/mL lysozyme (Coolaber) for 30 min at 37°C, sonicated for 10 min on ice (150 W, 5 s on/off), and boiled for 10 min. Lysates were subjected to 12% SDS-PAGE and then analyzed by Western blotting using mouse anti-His monoclonal antibody (MAb) (TransGen Biotech) as the primary antibody and horseradish peroxidase (HRP)-conjugated goat anti-mouse IgG (Proteintech, Rosemont, IL, USA) as the secondary antibody.

Physiological indexes measured for recombinant WXD171- Csa1, -IsdB, -TraP, -MntC, and -Hla_H35L_ included growth status at various temperatures (16, 23, 30, 37, 42, 49, and 56°C), pH values (2.0, 3.0, 4.0, 5.0, 6.0, 7.0, 8.0, and 9.0), and NaCl concentrations (0, 1, 3, 5, 7, 9, and 11%). Gastrointestinal environmental tolerance of recombinant *Lactobacillus* was assessed by the above-described methods in terms of tolerance of simulated gastrointestinal fluid and simulated intestinal fluid. The hereditary stability of recombinant strain detection was assessed as described by Tang’s group (66). In brief, recombinant strains were incubated in MRS containing chloramphenicol for 24 h at 37°C (20 generations) and serially transferred to culture, and plasmids were extracted to confirm the presence of recombinant plasmids.

### Immunization.

Recombinant *Lactobacillus* was cultured in MRS added with chloramphenicol until an OD_600_ range of 0.6 to 0.8 was reached, induced with 5 ng/mL nisin for 6 h, and washed with PBS, and the concentration was adjusted to 10^10^ CFU/mL. Mice were inoculated orally with 2 × 10^9^ CFU of bacteria or with PBS. Immunization protocol was performed during days 0 to 2, booster administration during days 14 to 16, and second-booster administration during days 28 to 30.

### Mouse model of S. aureus-induced pulmonary infection.

Mice vaccinated with a particular engineered *Lactobacillus* strain were anesthetized by intraperitoneal injection of ketamine-xylazine on day 35 and inoculated i.n. with S. aureus USA300 (5 × 10^9^ CFU/40 μL/mouse) to initiate pulmonary infection (Fig. 3A). Survival and health status were monitored and recorded during a 3-day period following challenge for mice in each group (*n* = 10). For bacteriological and histological analyses, PBS- and WXD171-IsdB-treated mice (*n* = 5) were inoculated on day 35 with S. aureus USA300 (5 × 10^8^ CFU/40 μL/mouse). At 48 h following challenge, right lungs were removed by thoracotomy, weighed, and homogenized. Serial dilutions of 100 μL tissue homogenate were placed on mannitol salt agar (MSA) plates (HuanKai Microbial) and incubated overnight at 37°C, and bacterial colonies were counted using a Scan 300 automated colony counter. Left lungs were fixed, paraffin-embedded, sectioned (4 to 6 μm), hematoxylin-and-eosin (H&E) stained, and subjected to histological evaluation by light microscopy (Olympus, Shinjuku, Japan).

### Mouse model of S. aureus-induced skin infection.

Mice (*n* = 5 per group) were anesthetized as described above, inoculated on day 35 with S. aureus USA300 (5 × 10^8^ CFU/50 μL/mouse) by dorsal subcutaneous (s.c.) injection, and monitored daily for mass and abscess formation for 28 days (Fig. 4A). Areas of abscesses and associated overlying dermonecrotic lesions were estimated using the standard formula: (π/2) × length × width. For counting of S. aureus CFU in skin abscess lesions, mice were sacrificed on day 2, abscesses were removed and homogenized in PBS, serially diluted samples (*n* = 5 per group) were grown on MSA plates at 37°C, and bacterial colonies were counted as described above. For histopathological analysis, skin tissues (*n* = 3 per group) were fixed, paraffin-embedded, sectioned (4 to 6 μm), H&E stained, and evaluated by light microscopy.

### Mouse model of S. aureus-induced systemic infection.

Mice (*n* = 5 per group) were anesthetized as above, and challenged on day 35 with S. aureus USA300 (1 × 10^7^ CFU in 100 μL PBS) by retro-orbital intravenous (i.v.) injection (Fig. 5A). Survival status was monitored and recorded during a 14-day period following challenge for mice in each group (*n* = 10). Most mice were monitored until 3 days postinfection; however, mice that lost >20% body weight were considered dead with regard to survival status assessment to avoid utilizing subjective criteria for moribund status (67). For weight monitoring and bacteriological and histological analyses, PBS-, antigen-free WXD171-Ctrl-, and WXD171-IsdB-treated mice were inoculated i.v. on day 35 with S. aureus USA300 (2 × 10^6^ CFU/100 μL/mouse). Signs of morbidity (weight loss, ruffled fur, hunched posture, paralysis, inability to walk, and inability to consume food or water) were monitored for 2 weeks postinoculation (*n* = 10 per group). To evaluate bacterial burden in various organs, 3 mice were sacrificed 1 or 3 days postinoculation, and organs were removed, weighed, and homogenized. Serial dilutions of 100 μL tissue homogenate were incubated overnight on MSA plates at 37°C, and bacterial colonies were counted as described above. For histopathological analysis (*n* = 3 per group), the indicated organs were removed and processed as described in “Mouse model of S. aureus-induced pulmonary infection.”

### ELISA for determination of IgG and sIgA levels.

Levels of IsdB-specific IgG in serum and sIgA in mucosa were determined by enzyme-linked immunosorbent assay (ELISA). For PBS- and WXD171-IsdB-treated groups, mice (*n* = 6) were selected randomly on day 35, and various samples were collected as follows. Sera were collected from the retro-orbital plexus, kept at 37°C for 2 h (to allow clotting), centrifuged, and stored at −80°C. Contents of small intestines were collected by squeezing out with sterile forceps, suspended in PBS (0.1 g/1 mL), and centrifuged (3,000 × *g* for 15 min), and the supernatant was stored at −20°C. PPs in small intestines and lungs were collected, snap-frozen in liquid nitrogen, and ground. Ground tissue (0.1 g) was suspended in 1 mL PBS and centrifuged (3,000 × *g* for 15 min), and supernatant was stored at −80°C. For the next processing step, 96-well Microlon ELISA plates were coated with 0.5 μg/mL purified IsdB protein (obtained from the Escherichia coli expression system constructed in our previous study) overnight at 4°C, washed three times with PBS containing 0.05% Tween 20 (PBST), blocked with 5% bovine serum albumin (BSA) for 2 h at 37°C, and washed three times with PBST. Samples (dilution ratios, 1:50 for sera and 1:10 for intestinal contents, PPs, and lungs) were added, incubated 2 h at 37°C, and washed five times with PBST; HRP-conjugated goat anti-mouse IgG or IgA (Proteintech) was added for 2 h at 37°C, and samples were washed five times with PBST. Color was developed using 3,3′,5,5′-tetramethylbenzidine (TMB) single-component substrate solution (Solarbio Science & Technology Co., Beijing, China), and absorbance was measured as OD_450_.

### Lymphocyte proliferation assay.

Spleens and PPs (*n* = 3 per group) were collected from vaccinated mice on day 35. Single-cell suspensions were isolated through a 100-μm nylon cell strainer and then depleted of red blood cells (RBC) using 1× RBC lysis buffer (Invitrogen). The cell suspension (100 μL; ~10^5^ cells/mL) was seeded in a 96-well plate, stimulated by purified IsdB protein (final concentration, 20 μg/mL; positive control, 10 μg/mL concanavalin A [ConA]; negative control, RPMI 1640 medium), and cultured for 72 h at 37°C in 5% CO_2_ incubator. Lymphocyte proliferation was measured with a CellTiter 96 AQueous One Solution cell proliferation assay (MTS) (Promega Corp.; Madison, WI, USA) using the absorbance at 490 nm. The stimulation index (SI) was calculated as (OD value of stimulated cells)/(OD value of unstimulated control cells) (68).

### Cytokine level assay.

Spleens, PPs, lungs, and intestines were collected (*n* = 5) on day 35 as described in “Mouse model of S. aureus-induced systemic infection.” The levels of the cytokines IL-17, IL-4, and IFN-γ in supernatant were assayed using an ELISA kit (R&D Systems, Minneapolis, MN, USA). Methods were essentially the same as for antibody detection.

### Flow-cytometric assay.

Vaccinated mice (*n* = 5 for each group) were sacrificed on day 35 and at 24 and 72 h postinoculation. Single-cell suspensions were prepared from PPs, spleens, and peripheral blood, and cells (2 × 10^6^) were incubated (4°C for 30 min) and stained using LIVE/DEAD fixable blue dead-cell staining kit (Invitrogen). Stained cells were treated with allophycocyanin (APC)-conjugated anti-mouse CD3 or CD11b antibody, fluorescein isothiocyanate (FITC)-conjugated anti-mouse CD4 or CD8 antibody, phycoerythrin (PE)-conjugated anti-mouse Ly6G antibody, or PE/Cy7-conjugated anti-mouse TCRγ/δ antibody (eBioscience), washed twice, and resuspended in flow cytometry buffer (PBS, 0.5% FCS, 0.1% sodium azide). Antibodies were diluted 1:100. Approximately 1 × 10^6^ events were acquired on a fluorescence-activated cell sorter (FACS), and data were analyzed using the FCS Express program (De Novo Software, Pasadena, CA, USA).

### Statistical analysis.

Data were analyzed using GraphPad Prism 8 (GraphPad, San Diego, CA, USA), and results from each experiment are presented as means with standard deviations (SD) or standard errors of the means (SEM) from three replicates, repeated three times. Kaplan-Meier survival analysis was performed, and comparisons between two groups were made using log-rank (Mantel-Cox) tests. Other data were analyzed by *t* test or one-way analysis of variance (ANOVA). Differences with *P* values of <0.05 or <0.01 were considered significant or highly significant, respectively.
